# Postsynaptic spiking determines anti-Hebbian LTD in visual cortex basket cells

**DOI:** 10.3389/fnsyn.2025.1548563

**Published:** 2025-02-17

**Authors:** Christina Y. C. Chou, Wouter J. Droogers, Txomin Lalanne, Eric Fineberg, Tal Klimenko, Hannah Owens, P. Jesper Sjöström

**Affiliations:** ^1^Centre for Research in Neuroscience, BRaIN Program, Department of Neurology and Neurosurgery, Research Institute of the McGill University Health Centre, Montreal General Hospital, Montreal, QC, Canada; ^2^Integrated Program in Neuroscience, McGill University, Montreal, QC, Canada; ^3^EphyX Neuroscience, Bordeaux, France

**Keywords:** inhibitory interneurons, plasticity, synapse, spike-timing dependent plasticity, visual cortex, action potential backpropagation, calcium imaging

## Abstract

Long-term plasticity at pyramidal cell to basket cell (PC → BC) synapses is important for the functioning of cortical microcircuits. It is well known that at neocortical PC → PC synapses, dendritic calcium (Ca^2+^) dynamics signal coincident pre-and postsynaptic spiking which in turn triggers long-term potentiation (LTP). However, the link between dendritic Ca^2+^ dynamics and long-term plasticity at PC → BC synapses of primary visual cortex (V1) is not as well known. Here, we explored if PC → BC synaptic plasticity in developing V1 is sensitive to postsynaptic spiking. Two-photon (2P) Ca^2+^ imaging revealed that action potentials (APs) in dendrites of V1 layer-5 (L5) BCs back-propagated decrementally but actively to the location of PC → BC putative synaptic contacts. Pairing excitatory inputs with postsynaptic APs elicited dendritic Ca^2+^ supralinearities for pre-before-postsynaptic but not post-before-presynaptic temporal ordering, suggesting that APs could impact synaptic plasticity. In agreement, extracellular stimulation as well as high-throughput 2P optogenetic mapping of plasticity both revealed that pre-before-postsynaptic but not post-before-presynaptic pairing resulted in anti-Hebbian long-term depression (LTD). Our results demonstrate that V1 BC dendritic Ca^2+^ nonlinearities and synaptic plasticity at PC → BC connections are both sensitive to somatic spiking.

## 1 Introduction

Activity-dependent synaptic plasticity such as LTP underlies circuit development and memory encoding in the brain (Bliss and Collingridge, 1993; Katz and Shatz, 1996; Malenka and Bear, 2004; Hensch, 2005). Synchrony of postsynaptic EPSPs and APs triggers LTP induction at excitatory to excitatory cell (E → E) synapses (Markram et al., 1997; Feldman, 2000). LTP, in turn, is thought to underlie the formation of Hebbian cell assemblies (Hebb, 1972).

In PCs, spikes initiate at the axon hillock and travel backwards into dendrites in the form of back-propagating APs, or bAPs (Sjöström et al., 2008), which indicate postsynaptic spiking to activated synapses (Stuart and Häusser, 2001). Yet bAPs in PC dendrites gradually fail with distance from the soma (Stuart et al., 1997; Vetter et al., 2001), which means the induction of synaptic plasticity depends critically on whether bAPs make it to the synapse or not.

At cortical E → E synapses, dendritic Ca^2+^ transients are critical determinants of plasticity (Sjöström et al., 2008). Classically, strong Ca^2+^ elevations trigger LTP, whereas smaller and more prolonged Ca^2+^ transients elicit LTD (Lisman, 1989; Lisman, 2001). However, long-term plasticity at synapses to (E → I) and from interneurons (I → E) is more diverse and not as well described (McFarlan et al., 2023). Yet, long-term plasticity at E → I and I → E synapses plays important functional roles, such as for visual cortex critical period plasticity (Hensch, 2005; Yazaki-Sugiyama et al., 2009), somato-dendritic integration (Udakis et al., 2020), and the stabilization of neuronal networks (Vogels et al., 2011).

Although E → E plasticity typically is Hebbian, long-term plasticity at E → I and I → E synapses often has diverse non-Hebbian properties (McFarlan et al., 2023). For instance, in the hippocampal CA1 region, PC → BC LTP is anti-Hebbian (Kullmann and Lamsa, 2007; Lamsa et al., 2007). Here, coincidence of postsynaptic EPSPs and APs elicits supralinear dendritic Ca^2+^ signals mediated by Ca^2+^-permeable AMPA receptors (CP-AMPARs) that instead of LTP trigger LTD (Camiré and Topolnik, 2014; Topolnik and Camire, 2019).

It is unclear if plasticity at neocortical PC → BC synapses is sensitive to postsynaptic somatic spiking, although the prior literature suggest that this may be the case. First, CP-AMPARs are specifically expressed at V1 PC → BC but not PC → PC connections (Lalanne et al., 2016), so are perfectly positioned to trigger PC → BC plasticity. Second, neocortical PC → BC CP-AMPARs appear to be key to postsynaptic Ca^2+^ influx (Goldberg et al., 2003). Finally, synchronous EPSPs and APs elicit dendritic Ca^2+^ supralinearities at neocortical PC → BC synapses (Sancho and Bloodgood, 2018). In agreement, anti-Hebbian LTD was reported at presumptive PC → BC synapses in somatosensory cortex (Lu et al., 2007). However, APs backpropagate decrementally in V1 BC dendrites (Goldberg et al., 2003; Sancho and Bloodgood, 2018), so it is not clear what effect, if any, somatic BC spiking should have on neocortical PC → BC plasticity.

Here, we provide evidence that APs backpropagate actively into L5 BC dendrites of developing V1 and reach PC → BC synapses. We found that synchronous but not asynchronous EPSP/AP pairing yielded Ca^2+^ supralinearities, suggesting that somatic BC spiking could influence PC → BC synaptic plasticity. In agreement, PC → BC synaptic plasticity was sensitive to the timing of postsynaptic spiking, so that synchronous but not asynchronous EPSP/AP pairing elicited anti-Hebbian LTD. We conclude that postsynaptic spiking determines anti-Hebbian PC → BC LTD in neocortical L5.

## 2 Materials and methods

### 2.1 Animals and ethics statement

All experiments were performed in accordance with the Policies and Guidelines of the *Canadian Council on Animal Care* and overseen by the Montreal General Hospital *Facility Animal Care Committee*. Animals were kept on a 12 h:12 h light/dark cycle. C57BL/6 wild-type (WT) mice were bred in house or obtained from Charles River Laboratories (Wilmington, MA, USA). Homozygous Emx1^Cre/Cre^ mice were obtained from Jackson laboratory (strain #005628; Bar Harbor, ME) (Gorski et al., 2002). Females and males were used for all experiments.

### 2.2 Viral infection

Adeno-associated viruses (AAVs) AAV9-CAG-DIO-ChroME-ST-P2A-H2B-mRuby3 (plasmid #108912; Addgene, Watertown, MA) and AAV9-mDlx-GFP-Fishell-1 (plasmid #83900, Addgene) were diluted to a titer of ~2.7e12 GC/mL in sterile phosphate buffer solution (PBS) (Thermo Fisher Scientific, Waltham, MA) and aliquoted. Aliquoted AAVs were kept at –80°C until use, at which time ChroME and mDlx AAVs were mixed at a 2:1 ratio. We carried out intracerebral AAV injections in neonatal (P0-P2) Emx1^Cre/Cre^ mice according to previously described descriptions (Kim et al., 2014). Mice were cryoanesthetized and placed in a stereotaxic frame. The head was leveled on the anterior–posterior and medial-lateral axes then fixed in place with the rubber-covered blunt end of stereotaxic frame ear bars. A 33-gauge needle attached to a 10 μL gas-tight syringe (Hamilton Instruments, Reno, NV) was used for injections. Instead of injecting into the lateral ventricles (Kim et al., 2014), we opted to inject directly into V1 to achieve higher expression levels. One V1 site was injected, at 0.00 mm anterior–posterior, 1.05–1.15 mm medial-lateral, with respect to lambda suture coordinates, and infusions of 0.2–0.3 μL were conducted at depths of 0.20 mm, 0.15 mm, and 0.10 mm below the pial surface, at a rate of 0.25 μL/min. The syringe was slowly retracted over 2 min to reduce the backflow of AAV mixture upon needle removal. Anesthetized mice were kept on a heating pad until movement was recovered then returned to their home cage.

### 2.3 Acute brain slice electrophysiology

During the light cycle, WT or injected mice aged P12-P25 were anesthetized with isoflurane and decapitated once the limb withdrawal reflex was lost. Brains were removed and immediately submerged in ice cold (~4°C) artificial cerebral spinal solution (ACSF) containing (in mM): NaCl, 125; KCl, 2.5; MgCl_2_, 1; NaH_2_PO_4_, 1.25; CaCl_2_, 2; NaHCO_3_, 26; Dextrose, 25; bubbled with 95% O_2_/5% CO_2_. Osmolality of the ACSF was adjusted to 338 mOsm with D-glucose, measured with Model 3,300 or Osmo1 osmometers (Advanced Instruments Inc., Norwood, MA). Dissection, incubation, and experiments were carried out in this solution unless otherwise stated. Ceramic blades (Lafayette Instruments, Lafayette, IN) mounted on a Campden Instruments 5000mz-2 vibrating microtome (Campden Instruments, UK) was used to cut 300 μm-thick near-coronal slices from V1, according to standard procedures (Abrahamsson et al., 2016). Brain slices were incubated at 36°C for 10 min then allowed to cool to room temperature. Experiments were varied out in ACSF heated to 33°C with a resistive inline heater (Scientifica Ltd., UK), with temperature recorded and verified offline. Recordings were truncated or discarded if temperature fell outside the 32–34°C range.

Whole-cell patch clamp and extracellular stimulation pipettes of 4–7 MΩ resistance were pulled from medium-walled capillaries on a P-1000 Flaming-Brown Micropipette Puller (Sutter Instruments, Novato, CA). Patch pipettes were filled with internal solution containing (in mM): KCl, 5; K-Gluconate, 115; K-HEPES, 10; MgATP, 4; NaGTP, 0.3; Na-Phosphocreatine, 10; and 0.1% w/v Biocytin, adjusted with KOH to pH 7.2–7.4 and with sucrose to 310 mOsm. Internal solution was supplemented with Alexa Fluor 594, 10–40 μm (Life Technologies, Waltham, MA). In Ca^2+^ imaging experiments, the internal solution was supplemented with 180 μM Fluo-5F pentapotassium salt (Life Technologies, Waltham, MA) (Yasuda et al., 2004). Patch pipettes used on BCs were also supplemented with 100 μM spermine (Millipore-Sigma, Burlington, MA) to account for dilution of polyamines from the cytoplasm (Kullmann and Lamsa, 2007) and to preserve depolarization-induced blocking of CP-AMPARs by polyamines (Bowie and Mayer, 1995; Donevan and Rogawski, 1995). Extracellular stimulation pipettes were filled with ACSF 40 μM Alexa Fluor 594 to aid in visualization.

Whole-cell recordings were obtained using BVC-700A amplifiers (Dagan Corporation, Minneapolis, MN) in current-clamp configuration. Current clamp recordings were filtered at 5–6 kHz and acquired at 10 kHz or 40 kHz using PCI-6229 boards (National Instruments, Austin, TX) with custom software (Sjöström et al., 2003) (available at https://github.com/pj-sjostrom/MultiPatch.git) running in Igor Pro 8 or 9 (WaveMetrics Inc., Lake Oswego, OR) on custom built computers (SL-DK-WS-PD-C236SAE-IF upgraded to quad-core Intel Core i7-6700, SuperLogics, Natick, MA). Series resistance, perfusion temperature, input resistance, and resting membrane potential were monitored online and assessed offline (see below). Series resistance was not compensated. Liquid junction potential (10 mV) was not accounted for.

Neurons were visualized and targeted for patch clamp with a LUMPlanFL N 40×/0.80 objective (Olympus, Melville, NY) using infrared video Dodt contrast on a custom modified Scientifica SliceScope (Buchanan et al., 2012). V1 was identified by the position of the dorsal hippocampal commissure and distinguished from the secondary visual cortex by the presence of L4. L5 PCs were identified by their large size, triangular shape, and prominent apical dendrites. BCs were identified by their smaller size and rounder shape. In some experiments, BCs were also targeted based on GFP expression visualized with 2P microscopy at 920 nm (see below).

To assess the electrophysiological characteristics of patched neurons for cell type identification, we injected 500-ms-long depolarizing current steps starting at −10 pA and ending at 200–600 pA, in 10 to 20-pA steps. The smallest current injection trace that elicited spikes, i.e., the rheobase trace, was used to measure spike width, heigh, and spike threshold. Instantaneous frequency was measured from the first two spikes in the rheobase trace. Accommodation was calculated as the ratio of instantaneous frequency for the first and last spike pairs in the rheobase spike train. If the rheobase trace did not have more than one spike, instantaneous frequency and accommodation were measured from next higher current injection after the rheobase.

Like before (Sjöström et al., 2001, 2003; Sjöström and Häusser, 2006), we relied on quality selection criteria. Input and series resistance were assessed with a 250-ms-long hyperpolarizing test pulse of −25 pA. Recordings with more than 30% change in input resistance or more than 8 mV change in resting membrane potential were discarded or truncated.

### 2.4 2P microscopy

2P microscopy as well as 2P optogenetics plasticity mapping (see below) was performed with workstations custom built from Scientifica SliceScope microscopes, as previously described (Buchanan et al., 2012). Using the same 40×/0.80 objective, 2P excitation was achieved using a Chameleon ULTRA II (Coherent, Santa Clara, CA) titanium-sapphire laser. Experiments characterizing Ca^2+^ supralinenarities in BC dendrites were conducted on a similar workstation equipped with a MaiTai BB (Spectraphysics, Santa Clara, CA) titanium-sapphire laser. The lasers were tuned to 820 nm for excitation of Alexa Fluor 594 and Fluo-5F. The Chameleon laser was tuned to 1,040 nm for 2P optogenetics plasticity mapping experiments. At each workstation, laser power was manually attenuated with polarizing beam splitter in combination with a half lambda plate (GL10-B and AHWP05M-980, Thorlabs Inc., Newton, NJ). A glass slide was used to reflect a fraction of the laser beam into a power meter (PM100A with S121C, Thorlabs) to monitor laser power.

The laser beam was first gated with a shutter (SH05/SC10, Thorlabs; or Uniblitz LS6ZM2/VCM-D1, Vincent Associates, Rochester NY) for safety. For 2P optogenetics plasticity mapping experiments, a galvanometric mirror (GVS011/M, Thorlabs) was subsequently installed in the beam path as a shutter capable of sub-millisecond precision. A pair of 3-mm galvanometric mirrors (6215H, Cambridge Technology, Bedford, MA) were used as beam scanners. Detectors were based on R3896 bialkali photomultipliers (Hamamatsu, Bridgewater, NJ). An FF665 dichroic and an FF01-680/SP-25 emitter (Semrock Inc., Rochester, NY) were used to collect fluorescence. An FF560-Di01 dichroic beam mirror (Semrock), an ET630/75 m (Chroma Technology, Bellow Falls, VT) red emitter, and an FF01-525/45–25 (Semrock) green emitter were used to separate red and green fluorescence. We collected the laser light after the spatial filter with an amplified diode (PDA100A-EC, Thorlabs) for laser-scanning Dodt contrast.

Images were acquired via PCI-6110 or PCIe-6374 boards (National Instruments) using ScanImage (Pologruto et al., 2003) version 3.7 (customized) or versions 2019 through 2022. In 2P optogenetics plasticity mapping experiments (Chou et al., 2024), images were acquired using in-house jScan software (https://github.com/pj-sjostrom/jScan) running in Igor Pro v8 or v9. Acquisition of imaging and electrophysiological data was synchronized using external triggering.

### 2.5 Action potential backpropagation in BC dendrites

We measured Ca^2+^ transients due to backpropagating action potentials (bAPs) in WT mice. Experiments were initiated at least 45 min after break-in to allow dye wash-in to distal dendrites. Action potentials were elicited in patched BCs via depolarizing current injections into the soma. Each current injection sweep started with a 250-ms-long 25-pA hyperpolarizing pulse to monitor membrane potential and input resistance, followed 500 ms later by five 5-ms-long 1.3-nA-pulses at 50 Hz. In the depolarized condition, 1.3-nA pulses were conducted in conjunction with somatic subthreshold depolarization (0.4 nA, 200 ms). Rest and depolarized sweeps were interleaved and repeated 10 times each with a 3 s inter-sweep interval.

Ca^2+^ responses via Fluo-5F were acquired by line scans (1 ms/line, 128 or 256 pixels/line, 1.84 or 3.67 pixels/μm) perpendicular to the dendrite at randomized dendritic locations ranging from the soma to the distal tip of the dendrite.

### 2.6 Postsynaptic Ca^2+^ transients in BC dendrites

We measured postsynaptic Ca^2+^ dynamics in WT mice. Experiments were initiated at least 45 min after break-in to allow dye wash-in to distal dendrites. After successful patching of a BC, the extracellular stimulation electrode was placed 10–60 μm away from the patched BC dendrites, as visualized by Alexa Fluor 594. EPSPs were elicited via biphasic voltage stimuli through the extracellular electrode connected to a BSI-950 Biphasic Stimulus Isolator (Dagan Corporation, Minneapolis, MN), while responses were obtained from the patched BC held in current clamp. Extracellular stimulation pulses were 100 μs in duration. Stimulation intensity and position of the stimulation pipette were adjusted to acquire EPSPs that had consistent and depolarizing amplitudes without hyperpolarizing currents. EPSPs were differentiated from direct stimulation of the patched neuron via the latency of depolarization, where EPSPs occurred 1–2 ms after stimulation onset.

Dendritic regions of interest (ROIs) were identified for Ca^2+^ imaging by comparing averaged Fluo-5F signal across 10 frame scans (64 × 64 pixels, 64 ms/frame) with and without extracellular stimulation (see Analysis section below). Once a ROI was chosen, synaptic pairing experiments were initiated.

In the synchronous group, pairing consisted of 5 extracellular stimulation pulses followed by 5 AP-inducing somatic current injection pulses (5 ms, 1.3 nA) at 50 Hz, offset by 10 ms. In the asynchronous group, pairing consisted of 5 somatic current injections followed by 5 extracellular stimulation pulses, offset by 90 ms. In each group, “EPSPs only,” “APs only,” and “both” conditions were interleaved and repeated 12–20 times each with an inter-sweep interval of 18 s. Ca^2+^ responses via Fluo-5F were acquired by 125 Hz frame scans (64 × 8 pixels, 8 ms/frame, 2.08 pixels/μm) over 2 s.

### 2.7 Analysis of Ca^2+^ dynamics

**Figure 1:**
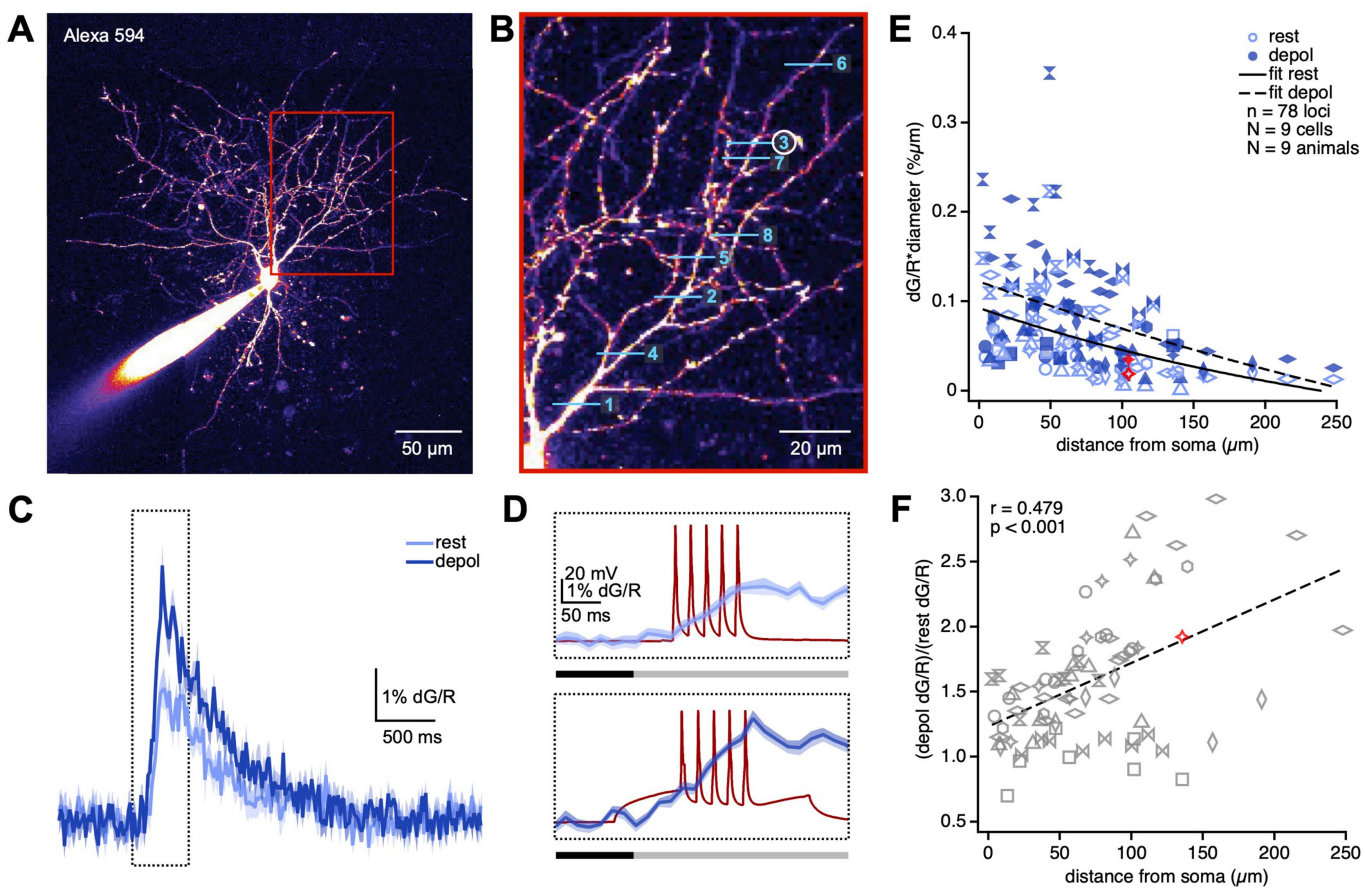
APs backpropagate decrementally but actively in BC dendrites. **(A)** Sample patched BC filled with Alexa 594 illustrate how dendritic processes were readily identified. Region in the red box is magnified in **(B)**. **(B)** We imaged Ca^2+^ transients using line scans at randomized locations along BC dendrites. In this sample experiment, line scans were acquired in the order denoted by the numbers. Ca^2+^ transients at line scan 3 (circled) is shown in **(C,D)**. **(C)** dG/R was recorded in response to a train of APs delivered while the BC was at rest (light blue) or depolarized (depol; dark blue). Sample Ca^2+^ transients were averaged across 20 sweeps at one dendritic location. The region in the dotted box is magnified in **(D)**. Shading denotes the SEM. **(D)** The dG/R integral was taken over a 275-ms-long window (grey bar) to measure dendritic Ca^2+^ transients due to bAPs (dark red traces). The 100-ms-long baseline period (black bar) was set to zero. **(E)** Ca^2+^ transients due to bAP diminished with distance from the soma (LMM, *p* < 0.0001). Depolarization boosted Ca^2+^ transients (rest, 72 ± 5; depolarized, 100 ± 6; LMM, *p* < 0.001), implying the involvement of active conductances in AP backpropagation. Different marker styles denote individual PCs. Red markers represent sample locus shown in **(B–F)**. Somatic depolarization boosted bAPs more with distance (Pearson’s *r* = 0.48, *p* < 0.001), suggesting active backpropagation. If boosting was passively mediated, the correlation ought to be non-existent or negative. Different marker styles denote individual PCs. Red marker indicates sample locus shown in **(B–E)**.

Quantification of Ca^2+^ signal magnitude was carried out using in-house LineScanAnalysis12 software running in Igor Pro (Sjöström and Häusser, 2006) (https://github.com/pj-sjostrom/LineScanAnalysis). Ca^2+^ signal magnitude was quantified as the ratio of the change in Ca^2+^-sensitive green fluorescence over change in Ca^2+^-insensitive red fluorescence (dG/R) from a 100–200 ms baseline period before stimulation onset. The dG/R in the ROI, was subtracted by dG/R in a background region, then integrated from 0 to 1,000 ms after stimulation onset (Yasuda et al., 2004). To account for surface to volume effects (Figure 1E), Ca^2+^ signal measurements were multiplied by the dendritic diameter (Sjöström and Häusser, 2006).

### 2.8 Induction of plasticity using 2P optogenetics

Emx^Cre/Cre^ mice injected with AAVs (described above) were used for 2P optogenetics plasticity experiments. In-house jScan software (https://github.com/pj-sjostrom/jScan) running in Igor Pro was used to create 2P optogenetic stimulation patterns and to acquire 2P imaging data, as previously described (Chou et al., 2024). Briefly, L5 BCs were patched as described above and a 2P image of mRuby fluorescence (512 × 512 pixels at 1.93 pixels/μm, average of 2 frames) was acquired at 1040 nm to identify mRuby-positive neurons as candidate presynaptic neurons for 2P stimulation. Fluorescent somata were semi-automatically selected and their xy coordinates were used to place Archimedean spiral trajectories (15 μm diameter, ~1 μm revolution spacing) over each cell body. This stimulation protocol has been shown to reliably elicit APs in ChroME-expressing neurons with millisecond temporal precision and near-single-cell spatial resolution (Chou et al., 2024).

Each candidate presynaptic neuron were spiral scanned 2 times (1,040 nm, 7 ms per spiral) at 30 Hz. Spiral scan bursts were separated by 500 ms across cells. Candidate presynaptic neurons in an ROI were thus sequentially stimulated 20 times every 20–30 s while the postsynaptic cell was whole-cell recorded in current-clamp mode. The 20 acquired sweeps were analyzed for EPSPs, as previously described (Chou et al., 2024). Synaptic responses were assigned to the corresponding presynaptic neuron.

Multiple connections were typically detected within a single ROI and LTP experiments were initiated in all identified presynaptic neurons. Pre-induction baseline sweeps consisted of 2 spiral scans at 30 Hz as described above, with presynaptic neurons spiral scanned sequentially every 500 ms. To monitor input resistance and a cell’s ability to spike, five 5-ms-long 1.3-nA pulses followed by a 250-ms-long −25 pA hyperpolarizing step were injected into the patched neuron 700 ms after the last spiral scan. Baseline sweeps were repeated 35–40 times, with an inter-sweep interval of 30–40 s, for a total of ~15 min, after which plasticity induction was initiated.

Plasticity induction consisted of 50-Hz pairing of 5 presynaptic spiral scans (as above) with 5 postsynaptic current pulses (5 ms, 1.3 nA), repeated 15 times every 20–30 s. In a ROI, each presynaptic neuron was randomly assigned different inductions: synchronous, asynchronous, or control. For synchronous, current pulses started 10 ms after spiral scans, so that evoked EPSPs and spikes coincided (Markram et al., 1997; Feldman, 2000; Sjöström et al., 2001). For asynchronous, the start of spiral scans trailed the start of current pulses by 90 ms, so that the first EPSP was evoked 10 ms after the last current injection in a train. In control, postsynaptic spiking was omitted. To ensure accurate relative timings, postsynaptic current pulses were temporally offset to account for EPSP latency, meaning timing differences are relative to EPSP onset (Feldman, 2000). Post-induction baseline sweeps were identical to pre-induction baseline sweeps, but were repeated for up to 1 h.

The magnitude of LTP was measured from the first EPSP of the 30-Hz paired pulse in baseline sweeps, by centering a ~ 1-ms window on the EPSP peak and subtracting an 8-ms period just prior to EPSP onset. To ensure that plasticity had enough time to express and stabilize, post-induction EPSP amplitude was measured starting 10 min after the induction, until experiments terminated. Paired-pulse ratio (PPR) was calculated as EPSP_2_/EPSP_1_. The change in PPR (ΔPPR) was calculated as PPR_after_ - PPR_before_. Coefficient of variation (CV) analysis was performed as previously described (Brock et al., 2020).

### 2.9 Cell morphometry

2P laser-scanning microscopy stacks (512 × 512 pixel slices at 1.68 pixels/μm separated by 1–3 μm) of each patched neuron was acquired at the end of the experiment. These were used for post-hoc verification of pyramidal and basket cell morphology. PCs were confirmed to have a large triangular soma and a prominent apical dendrite extending to the superficial cortical layers. BCs were confirmed to have small and rounded soma and lacked a prominent apical dendrite.

2P image stacks were contrast adjusted (Schindelin et al., 2012) and imported into Neuromantic (Myatt et al., 2012) for manual 3D tracing. Neocortical layers and image stack outlines were also manually labeled in Neuromantic. Quantification and statistical analysis of 3D reconstructions were carried out using in-house software qMorph (https://github.com/pj-sjostrom/qMorph) running in Igor Pro, as previously described (Buchanan et al., 2012; Zhou et al., 2021). Reconstructions were rotated around the soma so that the pial surface was straight up. Density maps were created by summing 2D Gaussians (*σ* = 25 μm, aligned on compartment XY centers and with amplitude proportional to compartment size) to form a smoothed morphology 2D projection. To average across reconstructions, individual density maps were peak normalized. To create symmetrical density maps, reconstructions were mirrored, but all comparisons were carried out on non-mirrored data. Jarvis walks were used to create convex hulls from 2D projections of individual reconstructions. Sholl analysis was carried out by aligning reconstructions on somata, converting to radial coordinates, moving outwards from somata in 6.5-μmm steps, and counting the number of compartments crossing a radius at hand. Sholl plots were averaged without normalization.

### 2.10 Identification of putative synaptic contacts

To find connected PCs and BCs, we relied on quadruple whole-cell recordings, which increases the yield by testing 12 possible connections in one go. Connectivity was verified by spiking each patched cell in sequence using five 5-ms-long current injection delivered at 30 Hz and monitoring for EPSPs in candidate postsynaptic neurons across 20 repeats or more. If cells were connected, we acquired image stacks after >45 min, to allow sufficient dye filling. Image stacks were used for neuronal reconstructions using Neuromantic, as described above. Putative synaptic contacts were identified in reconstructions axon and dendrites crossing within <2 μm and were confirmed visually in 3D image stack.

### 2.11 Statistics

Results are reported as mean ± standard error of the mean (SEM), unless otherwise stated. Ca^2+^ signals due to bAPs and presynaptic stimulation and LTP were compared between experimental groups using linear mixed models (LMMs) running in RStudio 2023.06.1.524 (Posit Software, Boston, MA). We used mixed models because individual measurements were often obtained from the same neuron or the same animal, meaning data points were not independent. Therefore, individual presynaptic neurons nested in individual animals were included as random effects in our analysis.

We fitted LMMs on bAP-induced Ca^2+^ signals using the lme() function from the nlme package (Pinheiro and Bates, 2000). Distance from soma, cell type (PC or BC), and condition (depolarized or rest) were included as fixed effects; distance from soma and individual cell ID were included as random effects. The lme() function uses the restricted maximum likelihood method to estimate LMM statistics. An F test was carried out to test the significance of categorical fixed effects (cell type and condition) using the fitted model as the argument in the anova() function. We found that cell type had no effect on bAP-induced Ca^2+^ signals and models (*p* = 0.64). We also found that the model which did not include cell type as a fixed effect returned a lower AIC value than the model which included cell type. For these reasons, we conducted statistical tests on the model that did not include cell type as a fixed effect. If fixed effects were significant at the *p* < 0.05 level, pairwise comparisons were carried out using the emmeans() function from the emmeans package (Lenth, 2023), which uses the Tukey method to adjust *p*-values for multiple comparisons.

We fitted LMMs on postsynaptic Ca^2+^ signals using the lme() function, after which an F test was carried out to test the significance of fixed effects — synchronous versus asynchronous spike timing and condition (“EPSPs,” “APs,” and “both”) — using the anova() function. If fixed effects were significant, pairwise comparisons were carried out using the emmeans() function.

We fitted LMMs on LTP measurements (EPSP_after_/EPSP_before_) and PPR (PPR_after_ – PPR_before_) using the lme() function. An F test was carried out to test the significance of fixed effects, i.e., spike timing.

## 3 Results

### 3.1 Action potential backpropagation in BC dendrites is decremental and active

In V1 as well as hippocampal BC dendrites, APs backpropagate decrementally (Goldberg et al., 2003; Camiré and Topolnik, 2014; Sancho and Bloodgood, 2018). A hallmark feature of propagation failure in PCs is that depolarization boosts bAPs (Stuart and Häusser, 2001; Sjöström and Häusser, 2006). We wondered if backpropagation was active in V1 L5 BC dendrites, in which case bAP-mediated Ca^2+^ transients should be sensitive to depolarization. To explore this, we targeted V1 L5 BCs for whole-cell recording. BC identity was verified *post-hoc* by spike pattern and morphometry (Supplementary Figure S1). As previously described (Markram et al., 2004; Gouwens et al., 2020), BCs spiking was characteristically non-accommodating with sub-millisecond spike half-width (Supplementary Figures S1L–O). BC neurites were generally restricted to ~150 μm radius from the cell soma (Supplementary Figure S1K) and chiefly remained within L5 (Supplementary Figures S1I,J), although V1 L5 BC axons occasionally ascended well into L2/3 (Supplementary Figure S1I), as previously described (Buchanan et al., 2012).

To visualize Ca^2+^ transients elicited by bAPs, patched BCs were loaded with the Ca^2+^ indicator Fluo-5F as well as the morphological dye Alexa 594 (Figure 1A). After allowing dyes to equilibrate for at least 25 min, we elicited 30-Hz trains of 5 APs while acquiring line scans at relatively random locations in the BC dendritic arbor (Figures 1B–D). We thereby characterized the spatial profile of Ca^2+^ transients elicited by bAPs with the BC in a resting versus a depolarized stated (Figures 1C,D; see Methods).

Due to scattering of laser light as well as of emitted fluorescence by brain tissue, line scans deeper into brain tissue yield less fluorescence. To account for varying depths of different line scan locations, we therefore measured Ca^2+^ transients as the change in green Fluo-5F fluorescence normalized to Alexa 594 red fluorescence (dG/R). To account for surface to volume effects, we multiplied dG/R measurements with the dendrite diameter at the line scan location (Sjöström and Häusser, 2006). To account for non-independence of measurements obtained from the same cell and the same animal, we relied on linear mixed model (LMM) statistics (see Methods) to determine whether Ca^2+^ transients depended on distance from soma and depolarization state.

In agreement with previous findings in V1 and hippocampal BCs (Camiré and Topolnik, 2014; Sancho and Bloodgood, 2018), we found that Ca^2+^ transients diminished with distance (Figure 1E), consistent with decremental AP backpropagation. We found that, similar to in PCs (Supplementary Figure S2) (Sjöström and Häusser, 2006), Ca^2+^ transients elicited by bAPs were boosted by somatic depolarization (Figure 1E). This boosting effect correlated positively with distance from the cell body, meaning more distal dendritic compartments were boosted more than proximal compartments (Figure 1F). This outcome suggests that bAPs reach farther when the BC is in a depolarized state, which in turn is in agreement with active AP backpropagation (Stuart et al., 1997; Stuart and Häusser, 2001; Vetter et al., 2001; Sjöström and Häusser, 2006). From this, we conclude that action potential backpropagation in BC dendrites is decremental as well as active. Based on our data, we cannot make claims about which active conductances are involved, other than that some of them must conduct Ca^2+^ ions.

### 3.2 PC → BC synapses are located close to the cell body

To influence long-term plasticity at PC → BC synapses, bAPs in BC dendrites must reach PC → BC synapses. Therefore, we investigated where along BC dendrites PC → BC synapses were found.

**Figure 2:**
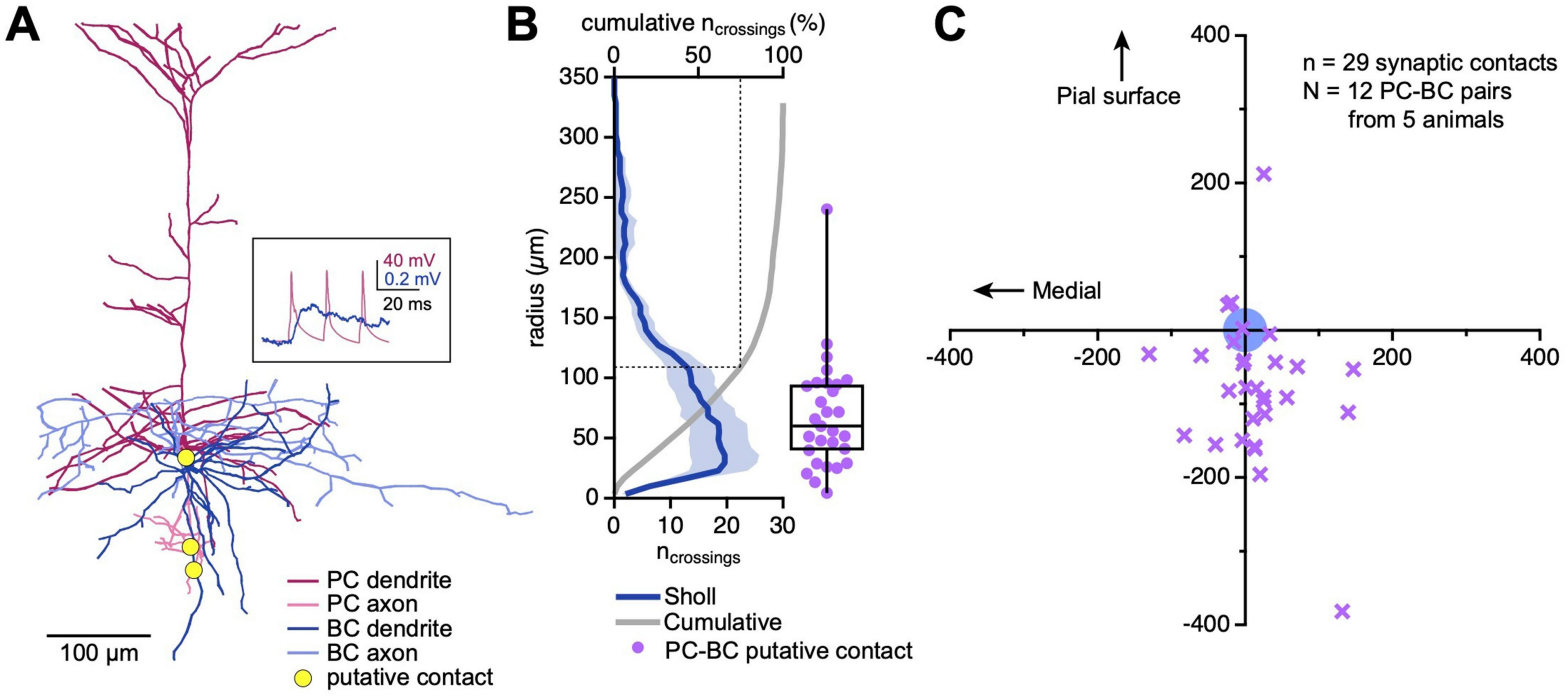
More than 75% of PC → BC synapses were located within 100 μm. **(A)** Sample reconstructed connected PC → BC pair. Yellow circles indicate contact points between the PC axon and the BC dendrite. Inset: EPSPs recorded from the BC (blue trace) in response to PC spiking (pink trace) are shown in the inset. **(B)** Sholl plot (blue trace) across *N* = 12 PC → BC pairs revealed that 75% of BC dendrites were restricted to within a radius of ~110 μm from the BC soma (dotted line). The majority of putative synaptic contacts (purple) were also found within this region. Dark blue: Sholl analysis mean, pale blue: Sholl analysis SEM, grey: cumulative normalized Sholl plot. Box plot: median, first, and third quartiles, with whiskers representing minimum and maximum. **(C)** Out of 29 putative synaptic contacts (purple markers), 25 were located below the BC soma (blue circle; putative contact y-coordinate −77 ± 20 μm vs. zero, *p* < 0.001, Wilcoxon rank).

We relied on recordings of synaptically connected PC → BC pairs that we acquired for a previous study (Lalanne et al., 2016). Patched cells were loaded with Alexa 594 dye for >45 min and 2P imaged in 3D, after which axonal and dendritic arbors were reconstructed offline (Figure 2A). We previously verified the accuracy of 3D reconstructions from 2P image stacks by comparing to confocal microscopy after biocytin histology (Blackman et al., 2014). Care was taken to ensure that neurites were not optically cut by 2P image stack boundaries. The resulting reconstructions of connected pairs of neurons were analyzed for putative synaptic contacts (see Methods).

We used Sholl analysis (Sholl, 1953) to measure the density of BC dendrites at increasing radii from the soma. We found a large majority of dendritic compartments within a ~ 150 μm radius from the cell body (Figure 2B). Similarly, 28 of 29 putative synaptic contacts were located in this region, on average ~ 69 μm from the BC soma, with 75% of contacts located within a 100 μm radius (Figures 2B,C). Taken together, these findings indicate that the majority of PC → BC synapses were located so close to the cell body that bAPs ought to reach them (Figures 1E,F). This in turn suggests that PC → BC synaptic plasticity might be sensitive to somatic spikes backpropagating into BC dendrites.

As an interesting aside, most of the identified putative synaptic contacts were located below the BC soma (Figure 2C). We previously found that L5 BCs preferentially received inputs from PCs located in upper L5 (Chou et al., 2024). Together, these two findings support a top-to-bottom asymmetry in the spatial distribution PC → BC connections, where presynaptic somata are located in upper L5 but synapse are made below. This could reflect the axon morphology of L5 PCs, which typically extend downwards (Figure 2A) (Gouwens et al., 2019).

### 3.3 BC dendrite Ca^2+^ supralinearities depend on spike timing

Since bAPs likely reach PC → BC synapses, we wondered if Ca^2+^ nonlinearities could result from different timings of pre-and postsynaptic spiking. A previous report used glutamate uncaging as a proxy for presynaptic activation and showed that the relative timing of pre-and postsynaptic spiking modulates dendritic Ca^2+^ dynamics in V1 BC dendrites (Sancho and Bloodgood, 2018). When glutamate uncaging preceded postsynaptic APs by 10 ms, Ca^2+^ supralinearities were observed, but when glutamate uncaging followed postsynaptic APs by 90 ms, Ca^2+^ signals summated sublinearly (Sancho and Bloodgood, 2018). We sought to replicate these findings.

**Figure 3:**
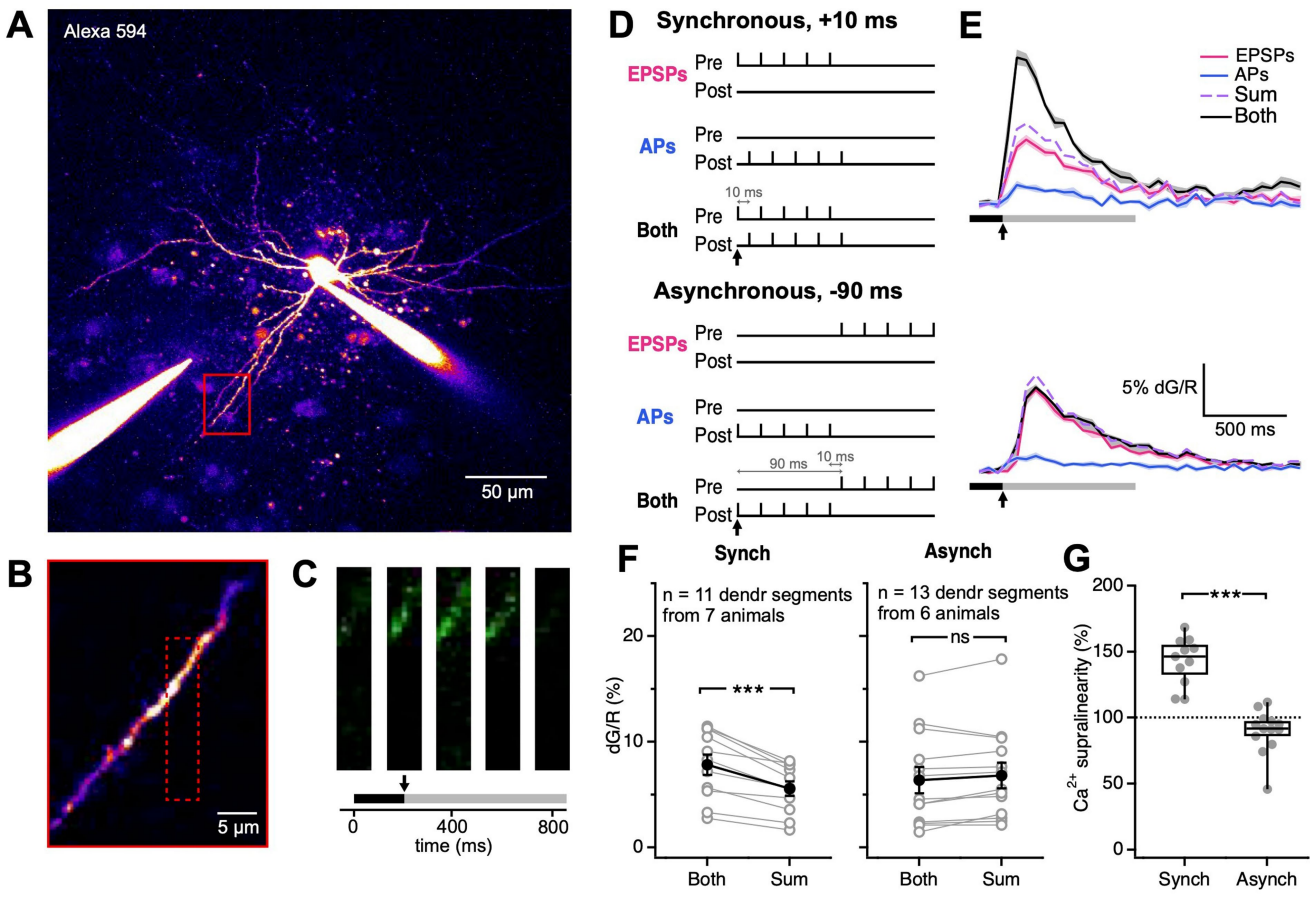
Synchronous EPSP-AP pairing elicited supralinear Ca^2+^ transients. **(A)** As indicated by this representative sample patched BC, the extracellular stimulation electrode was placed near the image BC dendrite (red box). **(B)** The dendritic region highlighted by the red box in **(A)** was targeted for frame scans. The dotted red box indicates the boundaries of the frame scan. **(C)** Ca^2+^ transients were detected by frame scans in response to extracellular stimulation and somatic current injection (onset denoted by the black arrow). The black bar represents the baseline period. The grey bar represents the period over which dG/R was integrated. The three images are sample movie frames positioned at their time points. **(D)** We paired pre-and postsynaptic activation in two different ways, synchronously or asynchronously. For each of the two ways, we used three conditions: presynaptic activity alone (“EPSPs”), postsynaptic activity alone (“APs”), or both (“Both”). Black arrows indicate the time of stimulation onset. **(E)** Ca^2+^ nonlinearity was assessed by comparing both to the arithmetic sum of EPSPs + APs. For synchronous pairing, postsynaptic activation followed presynaptic activation by Δt = 10 ms. For asynchronous pairing, postsynaptic activation preceded presynaptic activation by Δt = −90 ms. Solid line: mean. Shaded region: SEM. Black bar: baseline. Grey bar: integration period. Black arrows indicate stimulation onset, corresponding to black arrows in **(D)**. **(F)** Ca^2+^ supralinearity resulted from synchronous but not asynchronous pairing (*p* < 0.001, LMM; synchronous Both, 7.8% ± 1% vs. Sum, 5.6% ± 0.7%; *p* < 0.001; asynchronous Both, 6.4% ± 1% vs. Sum, 6.8% ± 1%; *p* = 0.14). Open circles: individual dendritic segments. Closed circles: mean ± SEM. **(G)** Synchronous and asynchronous pairing elicited different Ca^2+^ supralinearities in BC dendrites (Synch, 143% ± 5.4% vs. Asynch, 89.8% ± 4.6%; *p* < 0.001). Ca^2+^ supralinearity denotes the ratio dG/R_Both_ over dG/R_Sum_. Filled circles: individual dendritic segments. Box plot: median, first, and third quartile, with whiskers showing minimum and maximum.

We patched and loaded BCs with Fluo-5F, then used an extracellular stimulation pipette to activate excitatory afferents onto the patched BC (Figure 3A). We used 2P microscopy to identify Ca^2+^ responses to extracellular stimulation. Once a responsive dendritic region was found, we frame-scanned the region while evoking different activity patterns (Figure 3B). As before, Ca^2+^ transients were measured as the integral of dG/R (Figure 3C).

For synchronous pairings, pre-and postsynaptic activation were offset by 10 ms so that EPSP and APs were coincident (Markram et al., 1997; Feldman, 2000), whereas for asynchronous pairings, pre-and postsynaptic activation were offset by −90 ms, so that EPSPs and APs would miss each other (Figure 3D). To determine if coincident pre-and postsynaptic activity elicited Ca^2+^ non-linearities in the BC dendrite, we measured Ca^2+^ signals under 3 conditions: presynaptic activation only (“EPSPs”), postsynaptic activation only (“APs”), and both (“Both”; Figures 3D,E).

Synchronous pre-and postsynaptic activation resulted in Ca^2+^ transients greater than the arithmetic sum of either pre-or postsynaptic activation alone. In contrast, we did not observe Ca^2+^ nonlinearities in response to asynchronous stimulation (Figure 3F). Ca^2+^ dynamics in response to synchronous and asynchronous stimulation were thus strikingly different (Figure 3G).

### 3.4 Anti-Hebbian LTD at PC → BC synapses depends on spike timing

Dendritic Ca^2+^ nonlinearities have been linked to the induction of synaptic plasticity (Lamsa et al., 2007; Oren et al., 2009; Camiré and Topolnik, 2014). We therefore wondered if the dendritic Ca^2+^ nonlinearities we uncovered elicited plasticity at V1 PC → BC synapses.

To explore this possibility with a rapid throughput method, we searched for connected PC → BC pairs using 2-photon optogenetics. We expressed the opsin ChroME (Mardinly et al., 2018) in cortical excitatory neurons by intracerebral AAV injection in neonatal Emx1^cre/cre^ mice. We simultaneously tagged interneurons by co-injecting AAV expressing GFP under the mDlx-promoter (Dimidschstein et al., 2016). We previously showed that 2P optogenetics reliably drives spiking in PCs with single-cell spatial resolution and millisecond temporal precision (Chou et al., 2024).

**Figure 4:**
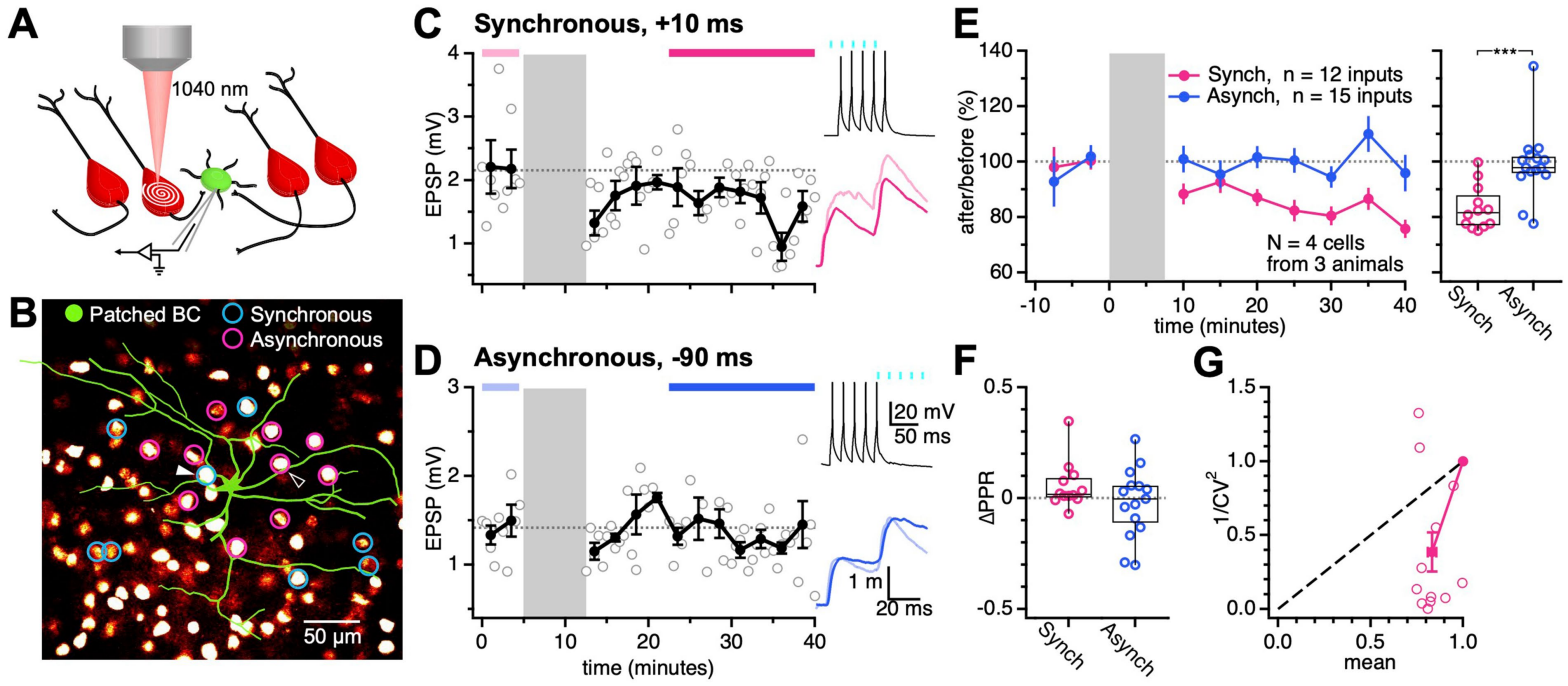
Synchronous EPSP-AP pairing elicited PC → BC LTD. **(A)** PC → BC synapses were identified using 2P optogenetics. A BC (green) was patched and monitored for EPSPs while surrounding ChroME-expressing PCs (red) were activated with femtosecond laser spiral scans. **(B)** Multiple ChroME expressing PCs (red fluorescence) were sequentially activated by 2P excitation to find connections onto the patched BC (green circle), an approach we call optomapping (Chou et al., 2024). Connected PCs were used in long-term plasticity experiments with synchronous Δt = 10 ms (blue circles) and asynchronous Δt = −90 ms pairings (pink circles). Sample PCs in **(C)** and **(D)** are indicated by triangles. **(C)** Synchronous pairing at this sample PC → BC synapse elicited LTD (after/before, 76%, *p* < 0.05). During the induction (grey), the presynaptic PC was activated by laser light (cyan rectangles) and the postsynaptic BC was activated by somatic current injections Δt = 10 ms later. Periods indicated by pink and red bars were used to quantify EPSPs (pink and red traces). **(D)** Asynchronous pairing at this sample PC → BC connection did not evoke detectable plasticity (after/before, 98%, *p* = 0.84). During the induction (grey), the presynaptic PC was activated (cyan rectangles) Δt = −90 ms after the postsynaptic BC. Periods indicated by light and dark blue bars were used to quantify EPSPs (light and dark blue traces). **(E)** Pooled across postsynaptic cells, synchronous pairing consistently evoked LTD (after/before, 83% ± 2%), while asynchronous pairing yielded no discernible plasticity (after/before, 99% ± 3%). Synchronous and asynchronous pairing yielded different long-term plasticity outcomes at PC → BC synapses (*p* < 0.001). **(F)** The change in PPR (ΔPPR) was indistinguishable for synchronous (0.056 ± 0.03) and asynchronous pairing (0.022 ± 0.04; *p* = 0.16). Open circles: individual connections. Box plot: median, first, and third quartile, with whiskers denoting minimum and maximum. **(G)** LTD caused 1/CV^2^ to decrease, suggesting that LTD was expressed presynaptically via reduced release (Brock et al., 2020). Open circles: individual connections. Filled circle: mean ± SEM.

To identify PC → BC connections, we patched BCs and stimulated surrounding PCs by spiral scanning them with a 1,040-nm femtosecond laser beam (Figures 4A,B; see Methods), an approach we call optomapping (Chou et al., 2024). This stimulation period was also used as the pre-induction baseline period for long-term plasticity measurements. Light-stimulated PCs that elicited EPSPs in the patched BC (Supplementary Figure S3) were next targeted for long-term plasticity induction. We targeted up to 14 connected PCs per patched BC (Figure 4B). During the induction, we paired activation of presynaptic PCs with activation of the patched postsynaptic BC, either with Δt = 10 ms so that peaks of EPSPs and APs were synchronous (Markram et al., 1997; Feldman, 2000), or asynchronously with Δt = −90 ms, so that EPSPs and APs did not overlap (Figures 4C,D; see Methods). The induction was followed by optomapping the same connections over a period lasting up to 1 h, to assess plasticity.

We compared PC → BC EPSPs before and after, which revealed that synchronous pairing triggered LTD (Figures 4C,E), whereas asynchronous pairing did not elicit detectable plasticity (Figures 4D,E). In other words, PC → BC plasticity depended on the relative timing of pre-and postsynaptic spiking, which demonstrated that postsynaptic spiking determined plasticity.

Although using optomapping for long-term plasticity provided high throughput, this method has to our knowledge not been use for this purpose before. We therefore sought to validate our findings. We performed synchronous and asynchronous plasticity induction protocols by stimulating excitatory afferents onto patched BCs using a monopolar stimulation electrode (Supplementary Figure S4). In agreement with optomapping, we found that synchronous activation of excitatory afferents and postsynaptic spiking induced LTD (Supplementary Figure S4A), whereas asynchronous pairing did not elicit detectable plasticity (Supplementary Figure S4B). Notably, we found that synchronous pairing triggered opposing plasticity outcomes for PC → BC and PC → PC connections, as the latter underwent LTP (Supplementary Figure S4D), as previously reported (Markram et al., 1997; Sjöström et al., 2001). These experiments thus validated the use of optomapping for long-term plasticity studies, while simultaneously highlighting that long-term plasticity depends on synapse type (Larsen and Sjöström, 2015; McFarlan et al., 2023).

To determine the locus of expression of PC → BC LTD, we analyzed PPR and CV (Blackman et al., 2013; Brock et al., 2020). For both optogenetic and electrical stimulation, PC → BC LTD did not appreciably alter PPR compared to the asynchronous group, potentially suggesting a postsynaptic locus of expression (Figure 4F; Supplementary Figure S4E). However, using both stimulation methods, PC → BC LTD reduced 1/CV^2^, suggesting reduced release (Figure 4G; Supplementary Figure S4F), because — assuming a binomial model of vesicle release — 1/CV^2^ is proportional to the release probability (Brock et al., 2020). This apparent discrepancy between PPR and CV analyses could be explained if 30-Hz stimulation did not sufficiently deplete the readily releasable pool of vesicles for PPR analysis to reveal a presynaptic change.

## 4 Discussion

Here, we report evidence to suggest that although bAPs attenuate with distance, they can reach PC → BC synapses on BC dendrites. We showed that synchronous but not asynchronous PC → BC spiking elicited Ca^2+^ supralinearities in BC dendrites. Synchronous PC → BC spiking also evoked LTD, while asynchronous activation did not elicit any detectable long-term plasticity. Taken together, our findings demonstrate that the spike timing of pre-and postsynaptic activity determines dendritic Ca^2+^ dynamics and LTD at L5 PC → BC synapses of developing V1. Our study also shows that computations in BC dendrites impact PC → BC synaptic plasticity.

### 4.1 Dendritic computations determine PC → BC synaptic plasticity

In PC dendrites, bAPs are key to coincidence detection in plasticity induction at PC → PC synapses (Stuart and Häusser, 2001; Nevian and Sakmann, 2006; Sjöström et al., 2008). The efficacy of AP backpropagation depends on cell type (Stuart et al., 1997), and if bAPs fail to reach distal dendrites, they cannot reliably contribute to the induction of plasticity (Sjöström and Häusser, 2006). Consequently, plasticity rules may thus differ at proximal and distal dendrites of the same neuron (Froemke et al., 2005; Letzkus et al., 2006; Sjöström and Häusser, 2006). Indeed, coincident pre-and postsynaptic activation has been shown to determine the sign of plasticity at hippocampal PC → BC synapses (Camiré and Topolnik, 2014).

Here, we showed that AP backpropagation — measured as transient increases in dendritic Ca^2+^ — attenuates with distance in BC dendrites, in line with prior literature (Goldberg et al., 2003; Sancho and Bloodgood, 2018). We also found that most PC → BC synapses were located close enough to the soma that bAPs should be able to reach them. On the other hand, synapses outside a ~ 100-μm radius from the BC soma may receive strongly attenuated bAPs. Therefore, plasticity that depends on coincidence of EPSP and APs is likely at proximal PC → BC synapses but less so at distal PC → BC synapses. It is important to note, however, that dendritic excitability changes during development (Sjöström et al., 2008), so this scenario may differ for BCs older than the developing ones we studied here.

The spatial reach of AP backpropagation depends on the distribution of dendritic ion channels as well as on dendritic morphology (Stuart et al., 1997; Vetter et al., 2001). In neocortical BC dendrites, I_a_-type K^+^ channels are known to limit the extent of AP backpropagation (Goldberg et al., 2003). Depolarization inactivates the I_a_-type K^+^ channels, which reduces repolarizing current and increases bAP amplitude (Migliore et al., 1999). In agreement, we found that subthreshold somatic depolarization boosted the amplitude and propagation of bAPs in V1 BC dendrites. Therefore, in addition to signaling spiking in the postsynaptic cell, bAPs in BC dendrites provide the synapse with contextual information regarding the conditions — such as coincident pre-and postsynaptic activation — that promoted reliable AP backpropagation.

### 4.2 Coincidence detection at PC → BC synapses

Excitatory synaptic input could provide the depolarization needed to boost bAPs (Goldberg et al., 2003). Indeed, several studies have shown that dendritic Ca^2+^ nonlinearities signal coincident pre-and postsynaptic activation at PC → BC synapses. While most studies in the hippocampus simultaneously paired theta-burst stimulation with postsynaptic depolarization or hyperpolarization for plasticity induction (Topolnik et al., 2005; Lamsa et al., 2007; Camiré and Topolnik, 2014; Hainmueller et al., 2014), a few studies in neocortex explored the dependence of dendritic Ca^2+^ nonlinearities on somatic spiking (Goldberg et al., 2003; Sancho and Bloodgood, 2018).

Spike-timing-dependent plasticity (STDP) is a biologically plausible coincidence-based learning rule at PC → PC synapses (Feldman, 2012). In its classical formulation, the relative timing of pre-and postsynaptic activation can indicate causality — when an EPSP arrives a few milliseconds before postsynaptic spiking, the EPSP was causally related to successful postsynaptic activation, and is rewarded by LTP (McFarlan et al., 2023). The opposite temporal ordering is termed acausal (McFarlan et al., 2023) and typically yields LTD (Markram et al., 1997; Feldman, 2000; Sjöström and Nelson, 2002). Causal and acausal spike timings generate different dendritic Ca^2+^ dynamics, which facilitates long-term plasticity (Kampa et al., 2006; Nevian and Sakmann, 2006).

In agreement with previous results (Goldberg et al., 2003; Sancho and Bloodgood, 2018), we observed Ca^2+^ supralinearities in V1 BC dendrites in response to pre-before-post synchronous spiking. Post-before-pre asynchronous spiking, however, did not elicit dendritic Ca^2+^ nonlinearities in our hands. Although we did not exhaustively explore different spike timings, our results suggest that spike-timing-dependent dendritic Ca^2+^ nonlinearities may underlie STDP at PC → BC synapses.

In the hippocampus, Ca^2+^ dynamics in BC dendrites determines PC → BC long-term plasticity (Lamsa et al., 2007; Oren et al., 2009; Camiré and Topolnik, 2014). In particular, Ca^2+^ dynamics in BC dendrites and the sign of hippocampal PC → BC plasticity is determined by whether postsynaptic spiking is sub or suprathreshold (Camiré and Topolnik, 2014; Topolnik and Camire, 2019). This view on plasticity is arguably distinct from the STDP paradigm. However, there is an interesting congruence in V1 and the hippocampus, where causal plasticity paradigms elicited supralinear Ca^2+^ dynamics. We also observed anti-Hebbian LTD at V1 PC → BC synapses, in line with non-classical forms of PC → BC plasticity observed in the hippocampus. Although we did not find anti-Hebbian LTP for subthreshold PC → BC activation, we would need to more exhaustively explore the parameter space to exclude its existence.

### 4.3 Optogenetics for high-throughput mapping of long-term plasticity

To assess the functional significance of spike-timing-dependent dendritic Ca^2+^ nonlinearities, we combined 2P optogenetics and whole-cell patch, a high-throughput technique we called optomapping (Chou et al., 2024), to conduct STDP experiments at PC → BC connections. Optomapping allowed us to simultaneously measure plasticity at up to a dozen connections in one go, which represents a dramatic increase in throughput compared to state-of-the-art paired patch techniques.

By conducting different spike timing pairings in the same postsynaptic neuron, our findings additionally demonstrated the synapse specificity of PC → BC plasticity. Coincidence detection thus occurs independently in different dendritic compartments, in line with previous descriptions of the highly compartmentalized nature of BC dendrites (Goldberg et al., 2003).

The diversity of the many different interneuron types gives rise to a great variety of E → I, I → E, and I → I synapse-type-specific plasticity rules (Larsen and Sjöström, 2015; McFarlan et al., 2024b), which collectively form a plasticitome (McFarlan et al., 2023). A long-standing problem for a comprehensive understanding of the cortical plasticitome (McFarlan et al., 2023) is thus the overwhelming diversity of interneuron types and the methodological difficulty associated with measuring all of their synapse-type-specific forms of short-term (Campagnola et al., 2022; Chou et al., 2024; McFarlan et al., 2024a) and long-term plasticity (McFarlan et al., 2024b). Here, we showcase a high-throughput plasticity mapping method that helps to resolve this enduring problem.

### 4.4 Caveats

One caveat of our study is that PPR and CV analyses disagreed on the locus of expression of plasticity — whereas CV analysis suggested presynaptic expression of anti-Hebbian LTD, the PPR was not changed after LTD, suggesting postsynaptic expression. This outcome was true for both optogenetic and electrical stimulation. This apparent discrepancy could be explained if the paired-pulse stimulation did not sufficiently deplete the readily-releasable pool of vesicles. Increasing the stimulation frequency and number of pulses would achieve better depletion, which could reveal a change in PPR. In this view, anti-Hebbian LTD would be presynaptic, as indicated by CV analysis. Retrograde endocannabinoid signaling has been shown to mediate LTD at PC → BC synapses in the hippocampus (Péterfi et al., 2012), suggesting that the same could hold true for PC → BC synapses in the neocortex. This supports the interpretation that expression is presynaptic, although more direct readout of presynaptic release — using, e.g., iGluSnFR (Marvin et al., 2013) or optical quantal analysis (Padamsey et al., 2019) — would be required to conclusively establish that expression is presynaptic.

During optomapping STDP experiments, we did not patch presynaptic neurons. While we recently demonstrated the reliability of photoactivating ChroME-expressing PCs (Chou et al., 2024), we did not directly monitor presynaptic spiking activity. We validated the optomapping method by repeating the STDP experiments using extracellular stimulation and obtained indistinguishable results. Furthermore, others have previously used optogenetics to measure long-term plasticity with no discernible adverse effects (Zhang and Oertner, 2007; Kohl et al., 2011; Shipton et al., 2014; Klavir et al., 2017; Kohl and Kätzel, 2017; Simmons et al., 2017; Polter et al., 2018; McFarlan et al., 2024b). Taken together, these observations lend strong support to our findings as well as to the use of optogenetics for the study of plasticity.

In agreement with findings from the hippocampus, we demonstrated anti-Hebbian LTD at PC → BC synapses for EPSPs paired with postsynaptic spiking (Lamsa et al., 2007; Camiré and Topolnik, 2014). Furthermore, post-before-pre asynchronous activation did not elicit changes in synaptic efficacy. However, unlike in hippocampus (Lamsa et al., 2007; Camiré and Topolnik, 2014), we did not observe anti-Hebbian LTP. One explanation for this discrepancy is that BCs in different brain regions follow different plasticity rules. Another not mutually exclusive explanation is simply that we would need to further explore the parameter space to find anti-Hebbian LTP.

Acute slice experiments suffer from artifacts such as hypoxia and lack of neuromodulation. Also, as typically is done with acute slice experiments, we relied on 2 mM external Ca^2+^ concentration, although it is ~1 mM in the intact brain (Markram et al., 2015), yet Ca^2+^ concentration is a key determinant of long-term plasticity (Inglebert et al., 2020; Chindemi et al., 2022). It is thus possible that the forms of plasticity we found here differ *in vivo*.

### 4.5 Future directions

Previous studies in the cat visual cortex demonstrated that proximal and distal dendritic regions of interneurons are selectively innervated by different afferent pathways (Freund et al., 1985; Freund and Meskenaite, 1992; Tamas et al., 1998). It is possible that the spatial distribution of afferent pathways on BC dendrites carry computational significance. We recently showed that V1 L5 BCs receive inputs from PCs in all neocortical layers (Chou et al., 2024). Here, we focused on connections from PCs in L5, but in the future, it would be interesting to explore whether PCs from other layers target specific regions on the BC dendrite.

Long-term plasticity in BCs has been shown to rely on CP-AMPARs, NMDARs, and mGluRs (Kullmann and Lamsa, 2007; Le Roux et al., 2013; Camiré and Topolnik, 2014; Hainmueller et al., 2014; Tran-Van-Minh et al., 2016). Plasticity outcomes at different synapses thus depended on receptor expression. An attractive candidate receptor for driving PC → BC STDP is the CP-AMPAR. CP-AMPARs are necessary for dendritic Ca^2+^ supralinearities and anti-Hebbian LTD in hippocampal BCs (Camiré and Topolnik, 2014). We previously demonstrated the synapse-type-specific expression of CP-AMPARs in V1 L5 BC dendrites (Lalanne et al., 2016; Lalanne et al., 2018). Pharmacology would reveal the receptors necessary for anti-Hebbian LTD at V1 PC → BC synapses.

Finally, we relied on one pre-before-post and one post-before-pre spike timing paradigm in this study. Some have argued that STDP is of limited biological relevance (Lisman and Spruston, 2005, 2010). At hippocampal PC → BC synapses, anti-Hebbian LTP was induced when presynaptic activation was paired with postsynaptic hyperpolarization (Lamsa et al., 2007). Plasticity may furthermore rely on neuromodulation (Huang et al., 2013). We are hopeful that recently developed high-throughput methods such as optomapping will aid researchers in exploring larger parameter spaces more efficiently. Our study thus does not paint the full picture of plasticity at neocortical PC → BC synapses but rather provides a starting point.

## Data Availability

The raw data supporting the conclusions of this article will be made available by the authors, without undue reservation.
