# Supplementary material for: Postsynaptic spiking determines anti-Hebbian LTD in visual cortex basket cells
Source: Front Synaptic Neurosci. 2025 Feb 17;17:1548563. doi: 10.3389/fnsyn.2025.1548563 (PMC11872923; doi:10.3389/fnsyn.2025.1548563)
Supplement: Supplementary file 1 [file Data_Sheet_1.PDF]

Supplementary Material

Supplementary figures

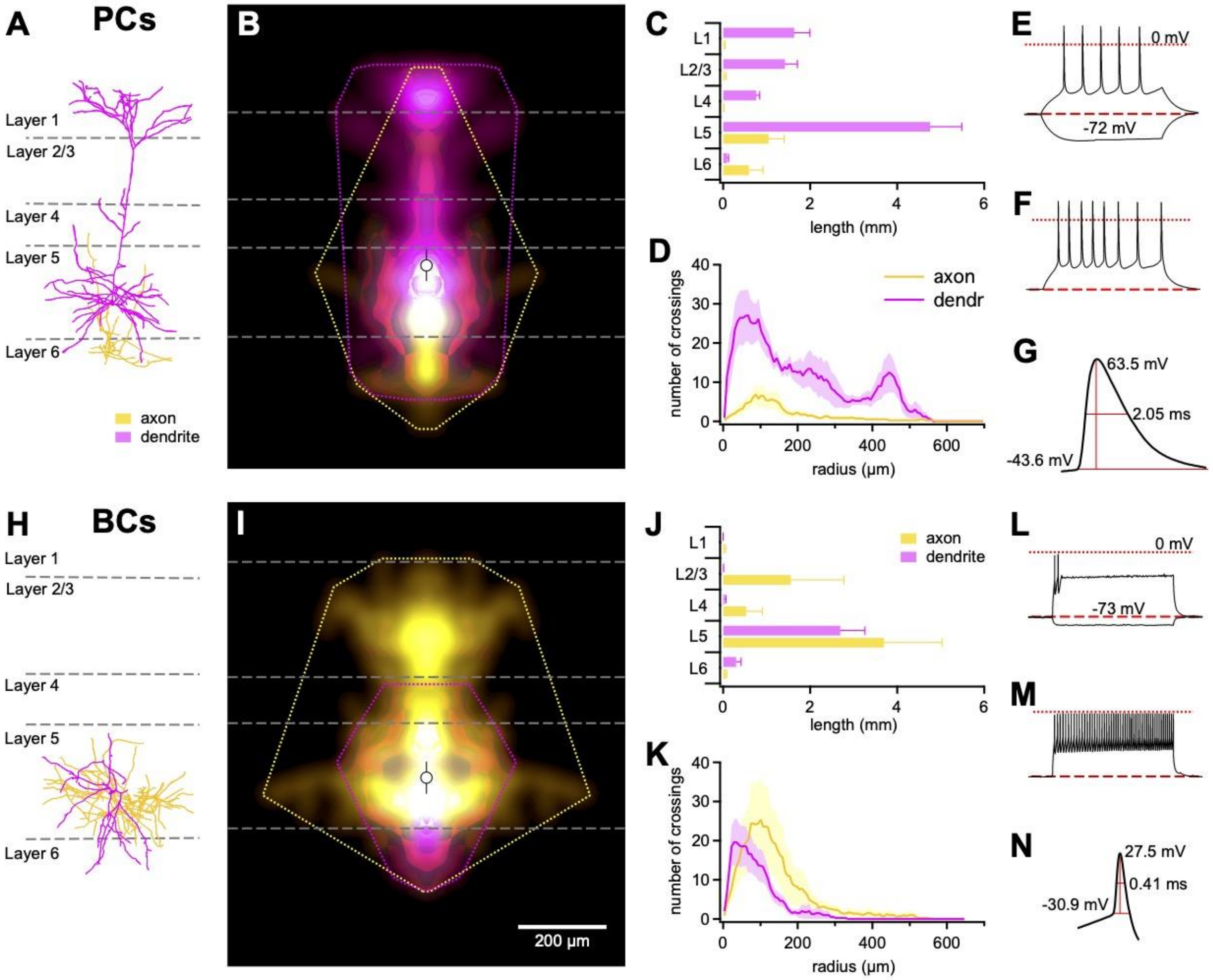

O

	PCs	BCs	p-value
n_{cells} ($n_{animals}$)	4 (4)	19 (16)	
Spike threshold (mV)	-40.95 ± 2.68	-31.48 ± 1.18	**
Spike height (mV)	54.67 ± 4.06	28.38 ± 1.55	***
Spike width at half height (ms)	2.41 ± 0.28	0.7 ± 0.03	**
Spike afterhyperpolarization (mV)	10.29 ± 3.21	-15.93 ± 0.82	***
Rheobase (nA)	0.04 ± 0.01	0.24 ± 0.03	***
Frequency (Hz)	41.5 ± 4.11	75.79 ± 8.36	**
Instantaneous frequency (Hz)	92.58 ± 7.96	100.3 ± 13	0.61796
Accommodation (%)	76.31 ± 27.2	-19.03 ± 8.58	**
Spike CV (%)	7.49 ± 6.02	5.07 ± 0.76	0.71623
Spike latency (ms)	10.25 ± 2.18	15.23 ± 4.23	0.30715
V_m (mV)	-66.28 ± 2	-69.99 ± 0.87	0.10353
R_{input} (MΩ)	436.17 ± 87.48	130.77 ± 12.94	*
Tau_m (ms)	40.04 ± 4.42	12.56 ± 0.78	**

Supplementary Figure 1. Distinct morphology and electrophysiology of L5 PCs and BCs

(A and B) L5 PCs have a prominent apical dendrite, which branches extensively in L1. A sample L5 PC reconstruction is shown in (A). Compartment density heatmap for eight L5 PCs is shown in (B). Heat maps are centered vertically on the boundary between L4 and L5.

(C) Layer-specific branching revealed that L5 PC dendrites ascend to superficial layers, including L1. The majority of L5 PC axons were distributed in L5 and L6.

(D) Sholl analysis indicated that L5 PC dendrites can be found >500 µm from the soma.

(E-G) L5 PC spike trains were accommodating, spikes generally reached a peak greater than 0 mV, and spike half-widths were >1 ms. (E) shows the rheobase trace and response to a hyperpolarizing current step from a sample L5 PC. In (F), the same L5 PC was stimulated with a rheobase +20 pA current pulse. A sample AP from the rheobase trace in (E) is shown in (G). The spike threshold, half-width, and amplitude are indicated.

(H and I) L5 BC dendrites and axons are mostly restricted to L5. A sample L5 BC reconstruction is shown in (H). Compartment density heatmap for eight L5 BCs is shown in (I). Heat maps are centered vertically on the boundary between L4 and L5.

(J) Layer-specific branching revealed that L5 BC dendrites were largely restricted to L5, with some branching in L6. Most L5 BC axons were also restricted to L5, however some BC axons extended to superficial layers.

(K) Sholl analysis indicated that the majority of L5 BC axons and dendrites are be found >200 µm from the soma.

(L-N) L5 BC spike trains were non-accommodating, spikes generally did not reach past 0 mV, and spike half-widths were <1 ms. (L) shows the rheobase trace and response to a hyperpolarizing current step from a sample L5 BC. In (M), the same L5 BC was stimulated with a rheobase +20 pA current pulse. A sample AP from the rheobase trace in (L) is shown in (N). The spike threshold, half-width, and amplitude are indicated.

(O) L5 PCs and BCs have distinct electrophysiological characteristics. L5 PCs have lower spike threshold, rheobase, and spike frequency compared to L5 BCs. Spike height, spike half-width, spike afterhyperpolarization, accommodation, R_{input}, and Tau_m were greater in L5 PCs than L5 BCs. P-values are indicated in the figure.

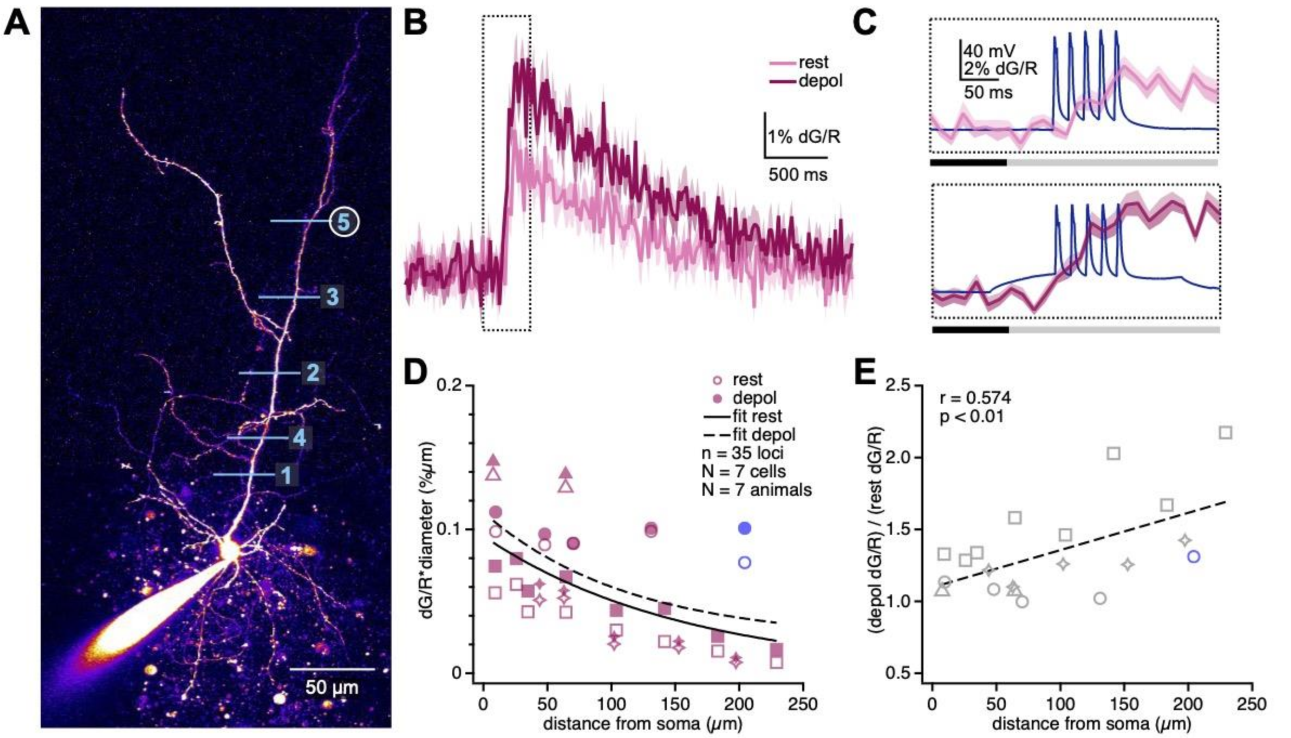

Supplementary Figure 2. APs backpropagate decrementally but actively in PC dendrites

(A) The apical dendrite can be readily identified in patched PCs. Here we show a max intensity projected 2P image stack of a sample PC patched and filled with Alexa 594. Line scans were acquired in the order denoted by the numbers. Ca^{2+} transients at line scan 5 (circled) is shown in (B) and (C).

(B) dG/R was recorded in response to a train of APs delivered while the PC was at rest (light pink) or in a depolarized state (dark pink). Here we show sample Ca^{2+} transients averaged across 20 sweeps at one dendritic line scan location. Shading denotes the SEM of the average Ca^{2+} signals. The region in the dotted box is magnified in (C).

(C) The dG/R integral was taken over a 275-ms-long window (grey bar) to measure dendritic Ca^{2+} transients due to bAPs (dark blue traces). The 100-ms-long baseline period (black bar) was set to zero.

(D) bAP-induced Ca^{2+} transients attenuated with increasing distance from the soma (n = 20 loci, N = 4 cells; LMM, $p < 0.0001$). Depolarizing the soma provided a boosting effect for bAP induced Ca^{2+} transients (rest, 72 ± 7 %µm; depolarized, 90 ± 8 %µm; LMM, $p < 0.001$). Different marker styles denote individual PCs. Blue markers represent sample locus shown in (B) and (C).

(E) Depolarization induced bAP boosting increased with distance from the soma. Different marker styles denote individual PCs. Blue marker represent sample locus shown in (B) and (C).

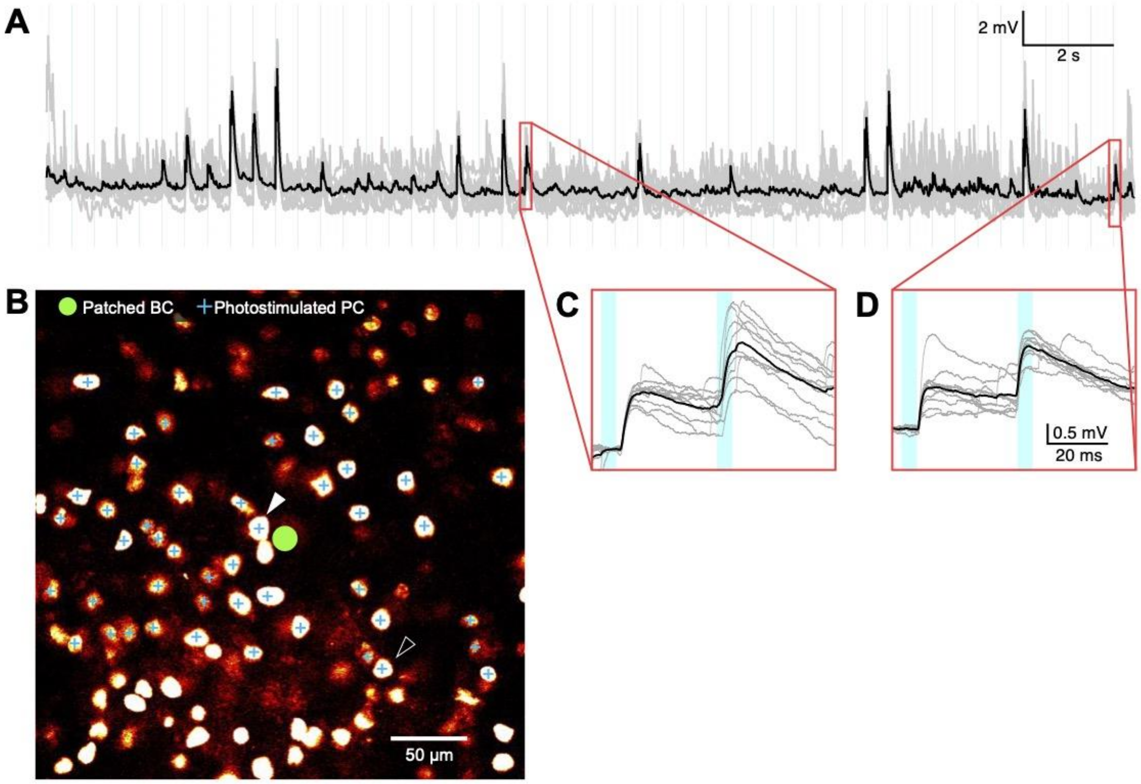

Supplementary Figure 3. Photostimulating PCs elicited EPSPs in the patched BC

(A and B) In this sample experiment, we whole-cell recorded from a current clamped BC while photostimulating 48 surrounding ChroME-expressing PCs. In (A), 10 BC response sweeps (grey) were averaged (black). Blue bars represent the onset of photostimulation on each PC. Only one laser pulse is depicted for each stimulation locus for clarity. Red insets correspond to EPSPs shown in (C) and (D). A 2PLSM image, taken at 1040 nm, of the photostimulated field of view is shown in (B). Photostimulated PCs are labeled with blue crosses. The filled triangle indicates the PC that elicited the EPSPs in (C). The open triangle indicates the PC that elicited the EPSPs in (D).

(C and D) When presynaptic PCs were photostimulated, EPSPs can be recorded in the patched BC. Blue bars represent laser pulses. Ten response sweeps (grey traces) were averaged (black trace).

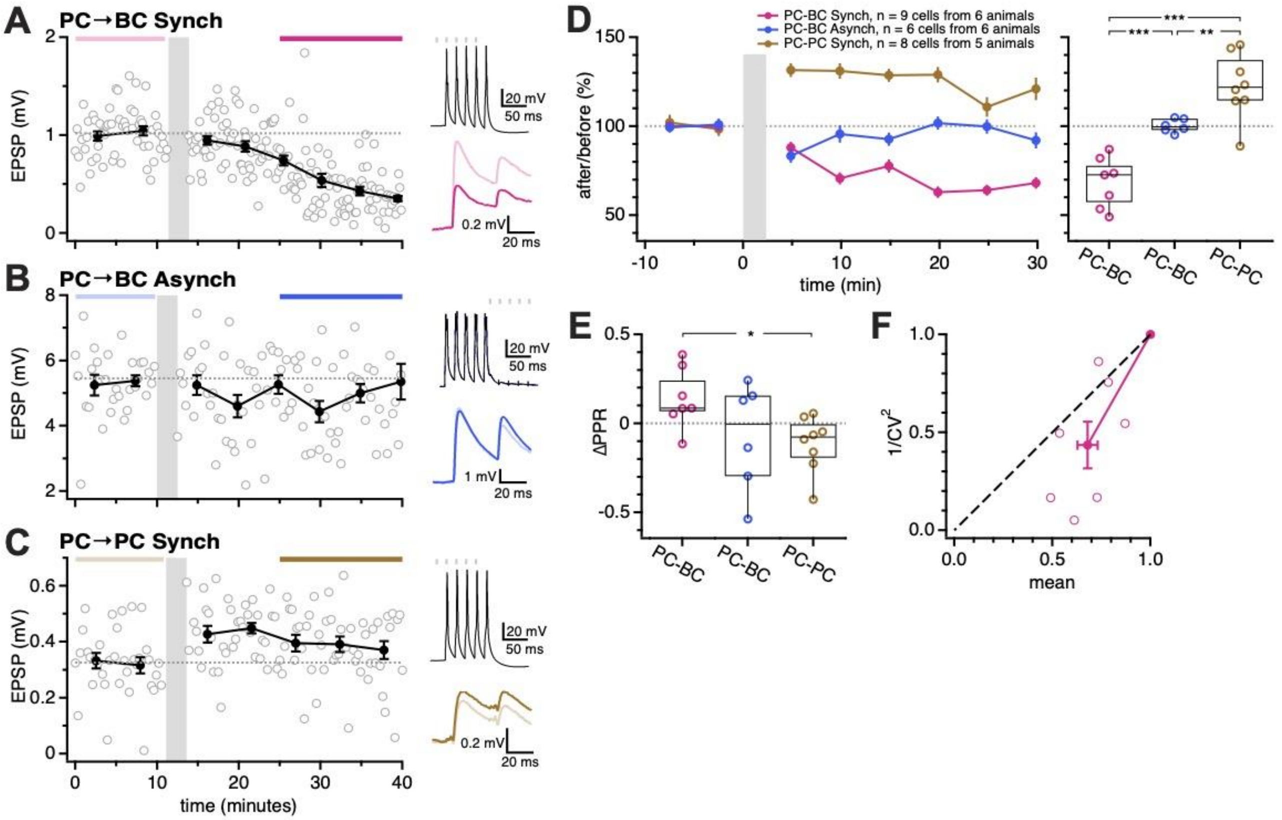

Supplementary Figure 4. Extracellular stimulation yielded similar PC→BC plasticity rules as optomapping

(A) Synchronous pairing at this sample PC→BC synapse elicited LTD (after/before, 49%, $p < 0.001$). During the induction (grey), excitatory afferents were activated by extracellular stimulation (black rectangles) and the postsynaptic BC was activated by somatic current injections t = 10 ms later. Periods indicated by pink and red bars were used to quantify EPSPs (pink and red traces).

(B) Asynchronous pairing at this sample PC→BC synapse did not evoke detectable plasticity (after/before, 95%, $p = 0.45$). During the induction (grey), excitatory afferents were activated by extracellular stimulation (black rectangles) and the postsynaptic BC was activated by somatic current injections t = -90 ms later. Periods indicated by light and dark blue bars were used to quantify EPSPs (light and dark blue traces).

(C) Synchronous pairing at this sample PC→PC synapse elicited LTP (after/before, 120%, $p < 0.01$). During the induction (grey), excitatory afferents were activated by extracellular stimulation (black rectangles) and the postsynaptic PC was activated by somatic current injections t = 10 ms later. Periods indicated by light and dark brown bars were used to quantify EPSPs (light and dark brown traces).

(D) Pooled across postsynaptic cells, we observed anti-Hebbian PC→BC LTD in response to synchronous pairing (after/before mean ± SEM, 68% ± 5%), no plasticity in response to asynchronous PC→BC pairing (after/before, 100% ± 2%), and Hebbian LTP in response to PC→PC synchronous pairing (after/before 120% ± 6%). The successful induction of PC→PC LTP with the same protocol demonstrates that PC→BC LTD is not a consequence of LTP failure. LMM statistics revealed that synchronous and asynchronous pairing of excitatory afferent activation and somatic APs at inputs onto BC and PCs yielded different plasticity outcomes (ANOVA, $p < 0.001$; PC→BC

synch vs. PC→BC asynch, $p < 0.01$; PC→BC synch vs. PC→PC synch, $p < 0.001$; PC→BC asynch vs. PC→PC synch, $p < 0.05$).

(E) The ΔPPR was indistinguishable between synchronous and asynchronous pairing onto BCs and PCs ($p = 0.12$).

(F) PC→BC LTD caused $1/CV^2$ to decrease, suggesting that LTD was expressed presynaptically via reduced release. Open circles: individual connections. Filled circle: mean ± SEM.
